# Identification, pathogenicity and effects of plant extracts on *Neopestalotiopsis* and *Pseudopestalotiopsis* causing fruit diseases

**DOI:** 10.1038/s41598-021-02113-5

**Published:** 2021-11-19

**Authors:** Angwara Darapanit, Nattawut Boonyuen, Wiphawee Leesutthiphonchai, Salilaporn Nuankaew, Onuma Piasai

**Affiliations:** 1 Department of Plant Pathology, Faculty of Agriculture, Kasetsart University, Bangkok, 10900 Thailand; 2 Plant Microbe Interaction Research Team, (APMT), Integrative Crop Biotechnology and Management Research Group, National Center for Genetic Engineering and Biotechnology (BIOTEC), 113 Thailand Science Park, Phahonyothin Road, Khlong Nueng, Khlong Luang, Pathum Thani, 12120 Thailand

**Keywords:** Microbiology, Molecular biology, Plant sciences

## Abstract

*Pestalotiopsis* and related genera, including *Neopestalotiopsis* and *Pseudopestalotiopsis* have damaged many plants for many decades; however, there is little available information about the fungi on tropical fruit in Thailand. This study isolated and characterized pestalotioid fungi on tropical fruit, investigated host specificity, and screened whether plant extracts could control the fungi. In total, 53 diseased fruit samples were sampled from eight types of fruit trees (jackfruit, rose apple, mangosteen, plum, snake fruit, rambutan, strawberry, and avocado). Based on morphological characteristics, 44 isolates were classified as belonging to pestalotioid taxa. Of these isolates, seven with distinct characteristics were selected for identification using molecular analysis, and six isolates were identified as *Neopestalotiopsis* and one as *Pseudopestalotiopsis*. In the cross-inoculation experiment, the isolates exhibited nonhost specificity and could infect at least two host plants. The isolates were used to screen for a potential biocontrol resource using six crude plant extracts (clove, ginger, lemongrass, mangosteen, roselle, and turmeric). All crude extracts except mangosteen could inhibit the growth of *Neopestalotiopsis* and *Pseudopestalotiopsis*. Application of crude plant extracts could be a potential treatment to control these diseases on tropical fruit.

## Introduction

*Pestalotiopsis *sensu lato comprises three genera—*Neopestalotiopsis*, *Pseudopestalotiopsis*, and *Pestalotiopsis *sensu stricto—established through phytopathogenic analysis based on the large subunit of the nuclear ribosomal RNA (LSU) region, and asexual morphs^1, 2^. Asexual morphs in these genera produce acervuli consisting of fusoid or ellipsoid to subcylindrical conidia with five cells, including an apical cell with appendages, a basal cell with an appendage, and three median cells positioned together with distinct colors^1, 3^.

*Neopestalotiopsis*, *Pseudopestalotiopsis*, and *Pestalotiopsis* infect a wide host range and currently cause problems worldwide^4–15^. The common symptoms of these pathogens include fruit rot, leaf blight, leaf spot, stem rot, grey blight, scabby canker, and post-harvest rot^1, 16, 17^. Examples of important plant pathogenic species are *Pestalotiopsis clavispora*, *P. furcta*, *P. versicolor*, *P. virgatula*, *Neopestalotiopsis piceana*, *N. samarangensis*, *Pseudopestalotiopsis camelliae sinensis*, and *Ps. indica*^18–20^.

*Pestalotiopsis *sensu lato threaten fruit production in Thailand. Several taxa of *Pestalotiopsis*, such as *P. aeruginea*, *P. expaillatii*, *P. palmarum*, *P. pipericola*, and *P. theae*, cause postharvest fruit rot and leaf blight disease in pear, cacao tree, banana, black pepper, and tea plant^21^. *N. samarangensis* causes fruit rot of rose apple^18^.

Pathogenicity of *Pestalotiopsis *sensu lato on a host has not been well understood. Plant pathogenic fungi in these genera are generally considered as a secondary pathogen; however, the diseases caused by the fungi have become more severe, such as the recent outbreak of *Neopestalotiopsis* in strawberries in Florida^22^. *Pestalotiopsis *sensu lato from different tissues of the same host exhibited a variation of pathogenicity in different tissues^23^.

Crude plant extracts can be an alternative to control diseases and can reduce the need to use fungicides. Many plants such as medicinal plants produce antimicrobial compounds and inhibit the growth of plant pathogens^24, 25^. For example, turmeric plants produce curcumin, eugenol, and eugenyl acetate that are effective against bacteria and fungi^26–30^.

*Pestalotiopsis* and its related genera have been problems of tropical fruits in Thailand for decades; however, little knowledge of these fungal pathogens has been available. The aims of the present study were: (1) to explore the pestalotioid fungi causing diseases of tropical fruit in Thailand based on both morphological data and molecular phylogeny using multilocus sequences of the internal transcribed spacer (ITS), the β-tubulin gene, and the translation elongation factor 1 alpha gene (*tef1*); (2) to study host specificity; and (3) to investigate the effectiveness of plant crude extracts against the mycelial growth of these genera.

## Materials and methods

### Sample collection

**Figure 1:**
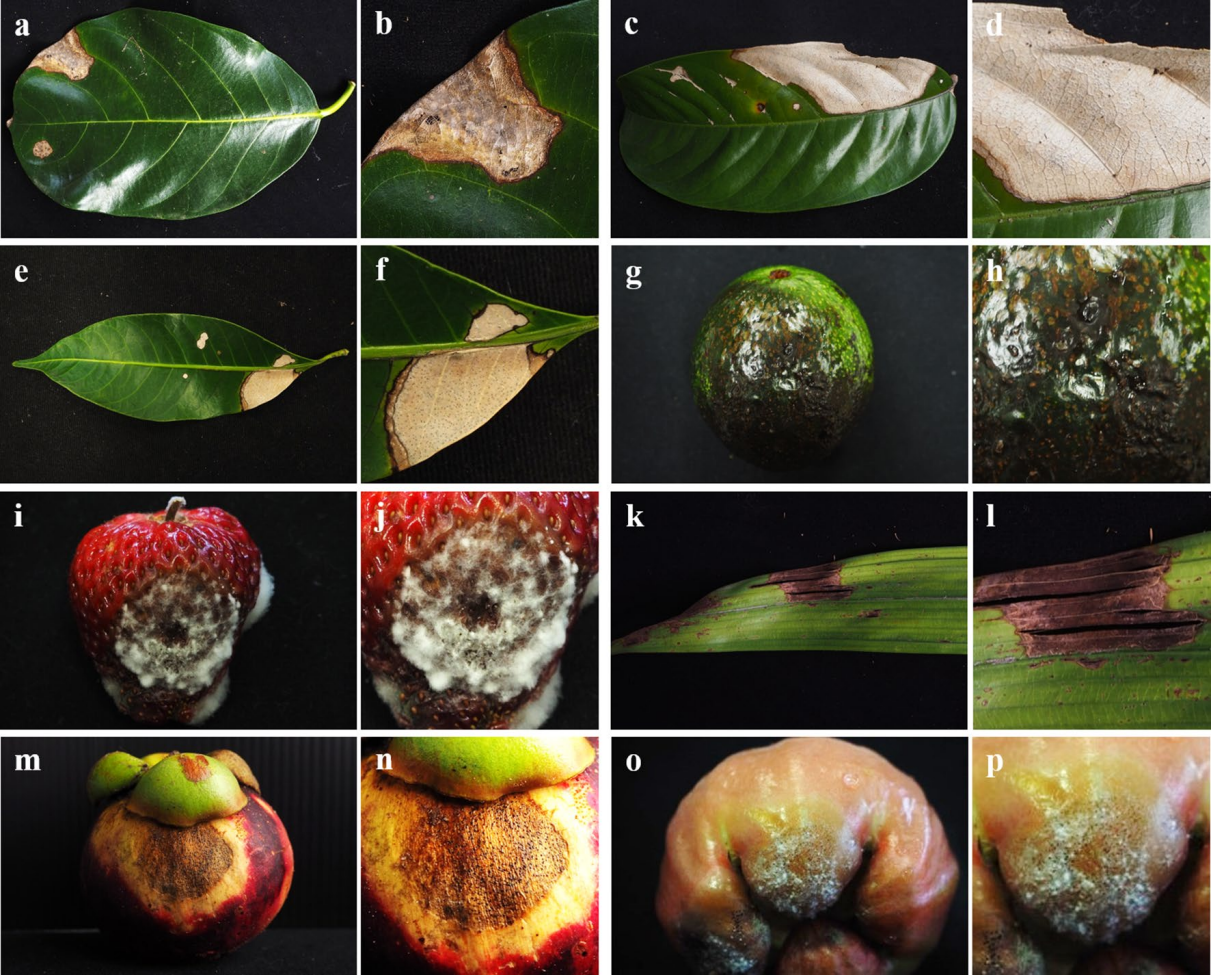
Symptoms of leaf blight and fruit rot on the original hosts caused by *Neopestalotiopsis* and *Pseudopestalotiopsis*. (**a**, **b**) Jackfruit leaves (*A. heterophyllus*); (**c**, **d**) rambutan leaves (*N. lappaceum*); (**e**, **f**) plum leaves (*B. macrophylla*); (**g**, **h**) avocado (*P. americana*); (**i**, **j**) strawberry (*F.* × *ananassa*); (**k**, **l**) leaves of snake fruit (*S. zalacca*); (**m**, **n**) mangosteen (*G. mangostana*); (**o**, **p**) rose apple (*S. samarangense*).

In total, 53 diseased plant samples showing leaf blight and fruit rot symptoms belonging to eight plant species were used, consisting of 5, 5, 13, 5, 6, 8, 6, and 5 samples from jackfruit (*Artocarpus heterophyllus* Lam.), rose apple (*Syzygium samarangense* [Blume] Merrill & L. M. Perry), mangosteen (*Garcinia mangostana* L.), plum (*Bouea macrophylla* Griffith), snake fruit (*Salacca zalacca* (Gaertn.) Voss), rambutan (*Nephelium lappaceum* L.), strawberry (*Fragaria* × *ananassa* Duchesne), and avocado (*Persea americana* Mill.), respectively (Fig. 1, Table 1). The samples were collected from private orchards in Chanthaburi, Phetchaburi, Nakhon Nayok, and Chiang Mai provinces, Thailand with the permission from each orchard owner between February 2017 and January 2018. The samples were recorded, photographed, and brought to the laboratory at the Department of Plant Pathology, Faculty of Agriculture, Kasetsart University (KU), Bangkok, Thailand for mycological isolation and morphological investigation. DNA extraction and PCR amplification were conducted in the National Center for Genetic Engineering and Biotechnology (BIOTEC), Pathum Thani, Thailand.

**Table 1:** Numbers of fruit diseases and fungal isolates in this study.

Host	No. of samples	Fungal isolates	No. of isolates
Jackfruit	5	PP036, PP037	2
Rose apple	5	PP039, PP040, PP041, PP042, PP043	5
Mangosteen	13	PP006, PP007, PP008, PP009, PP010, PP011, PP012, PP013, PP020, PP021, PP022, PP023, PP024, PP025, PP026	15
Plum	5	PP044	1
Snake fruit	6	PP030, PP031, PP032, PP033, PP034, PP035	6
Rambutan	8	PP014, PP015, PP016, PP017, PP018, PP019, PP027, PP028, PP029	9
Strawberry	6	PP038	1
Avocado	5	PP001, PP002, PP003, PP004, PP005	5
	53		44

### Fungal isolation and morphological data

The tissue transplantation technique was used to isolate fungal pathogens from the plant samples^31^. The tissue between healthy and infected parts was cut into 5 mm × 5 mm pieces, dipped in 0.25% sodium hypochlorite for 3 min, rinsed with sterile water, and dried with sterile tissue paper*.* The samples were placed in sterilized 9 cm Petri dishes, containing potato dextrose agar (PDA; Difco, Oxford, UK), incubated at 25 ± 2 °C for 3 days, and monitored daily. After incubation, the fungal isolates were examined and transferred to new PDA plates for identification. The macroscopic features of the fungi, such as colony color, colony diameter, and the exudates of each isolate, were recorded. Microscopic characteristics such as acervuli and conidial features were examined under a stereomicroscope (Olympus, Tokyo, Japan) and a compound microscope (Carl Zeiss, Jena, Germany) and compared with the features in identification keys and species descriptions^1, 8, 9, 18^.

### DNA extraction, PCR amplification, and sequencing

Total genomic DNA of fungal mycelia grown on PDA at 28 °C for 4 days was extracted using the protocol of Chuaseeharonnachai et al*.*^32^ and the CTAB method^33^. Fragments of the rDNA internal transcribed spacer or ITS1-5.8S-ITS4 region were amplified using the primer pairs ITS1 and ITS4 ^34^; the β-tubulin gene region was amplified using the primer pairs Bt2a and Bt2b ^35^; and the *tef1* gene region was amplified using the primer pairs EF1-526F and EF1-1567R^36^. Amplification and sequencing reactions, sequences analyses, and consensus of sequences were performed as described by Maharachchikumbura et al.^9^. The PCR amplicons were visualized by the staining of a 1% agarose electrophoresis gel. Sequencing was performed by Macrogen Inc. (Seoul, Korea). Generated sequences were deposited in NCBI under accession numbers MT628375–MT628381 for ITS sequences, MW776648–MW776654 for β-tubulin sequences, and MW776633–MW776638 for the *tef1* gene sequences.

### Phylogenetic analyses

The sequences generated in this study were compared with sequences retrieved from the GenBank which were reported^1, 37–39^. ITS, β-tubulin, and *tef1* gene sequence data were assembled using the alignment program BioEdit v7.2.5^40^, aligned using an online alignment program MAFFT version 7 (https://mafft.cbrc.jp/alignment/server/^41^) and edited manually as necessary using the alignment program BioEdit v7.2.5^40^. Phylogenetic tree estimation for each alignment was performed using Maximum Parsimony (MP), Maximum Likelihood (ML), and Bayesian Inference (BI). MP analyses were conducted using PAUP v. 4.0b10^42^, inferring trees with the heuristic search option with Tree-Bisection Reconnection branch swapping and 1000 random sequence additions. ML and bootstrapping analyses were conducted using RAxML-HPC v.8 on XSEDE^43^ in the CIPRES Science Gateway (http://www.phylo.org/portal2/^44^). The ML analysis was performed using the GTRGAMMA model with 1000 bootstrap iterations. The phylogenetic analyses were performed for Bayesian inference using MrBayes v. 3.2.7a^45^. Markov chain Monte Carlo sampling in MrBayes v.3.2.7a^45^ was used to determine the posterior probabilities. Four simultaneous Markov chains were run for 5,000,000 generations and trees were sampled every 1000th generation. Support values (ML bootstrap, ML-BS; MP bootstrap, MP-BS; BI posterior probability, BPP) were calculated for all analyses and the tree length (TL), consistency index (CI), retention index (RI), and the rescaled consistency index (RC) for the MP analysis generated under different optimality criteria. Phylogenetic trees were viewed and arranged using FigTree v1.4.2 (http://tree.bio.ed.ac.uk/software/figtree/).

### Pathogenicity tests

The fungal isolates were tested for pathogenicity in the original hosts of the pathogens based on the protocol of Phoulivong et al*.*^46^. The cross-inoculation experiment was conducted on host and other plants, consisting of mangosteen, avocado, rambutan, snake fruit, plum, strawberry, rose apple, and jackfruit. The selected fruits and leaves were cleaned with tap water and sterilized by dipping them into 70% ethanol for 3 min. The fruit was rinsed three times in sterile water and dried with sterile tissue paper. Each fruit was wounded with a sterile needle. Mycelial agar plugs from 7-day cultures were placed onto the surfaces of the leaf and fruit samples. Each of the treated samples was placed in a separate plastic box that served as a moist chamber and incubated at 25 ± 2 °C. All inoculated fruits and leaves were visually assessed daily and any symptoms were recorded. The fungi were re-isolated, and species comparison was performed to confirm the causal pathogen.

### Preparation of plant extracts

Fresh plants of turmeric (*Curcuma longa* L. (family Zingiberaceae)), ginger (*Zingiber officinale* L. (Zingiberaceae)), lemongrass (*Cymbopogon citratus* L. (Poaceae)), mangosteen (*Garcinia mangostana* L. (Clusiaceae)), roselle (*Hibiscus sabdariffa* L. (Malvaceae)), and clove (*Syzygium aromaticum* L. (Myrtaceae)) were purchased from the Talad Thai market, Khlong Luang district, Pathum Thani province, Thailand. Plants were washed thoroughly under running tap water and soaked in a 2% solution of sodium hypochlorite for 20 min, then rinsed thoroughly with sterilized water, and air-dried at 25 ± 2 °C for 4 h. The dried plant material was milled. To prepare crude extracts, about 500 g of finely ground plant material were submerged in 1000 ml ethanol solvent overnight at 25 ± 2 °C and passed through Whatman filter paper No. 1. The organic phase was evaporated in a rotary vacuum evaporator (BUCHI, Operation Manual Roatavapor^®^ R-210/215, Vacuum Pump V-700/710, Switzerland) under a pressure of 45 mbar at 40 °C, followed by storage at 4 °C until use.

### Determination of mycelial inhibition

The modified method of Cho et al*.*^47^ was used to determine the effect of the crude extracts on fungal growth. The center of Petri plates was inoculated with a mycelial plug obtained from a colony edge of each cultured fungi at age 7 days. The control was set up using blank agar plug plates (PDA mixed with distilled water). The fungicides (azoxystrobin + tebucolnazole, prochloraz, or captan) were prepared at recommended concentrations of 500, 1000, or 1500 mg/l, respectively*.* Five replicates of PDA-crude extract per isolate were incubated at 25 ± 2 °C, and radial growth was measured at 7 and 14 days. Inhibition levels (%) were calculated as (X–Y/X) × 100, where X and Y are the diameter of a fungal colony grown in a negative control plate and the diameter of a fungal colony grown in a plate containing a crude plant extract, respectively.

### Statistical analysis

The effect of different concentrations of crude extracts on the growth of fungi on PDA media was evaluated based on one-way analysis of variance (ANOVA), and significant differences were determined using Duncan’s multiple range test (P < 0.05). Conidial sizes were evaluated based on ANOVA and Wilcoxon signed-rank tests.

### Ethical approval

This article does not contain any studies with human participants or animals performed by any of the authors. Plant materials in this study complies with relevant institutional, national, and international guidelines, and legislation.

## Results

### Morphological and molecular identification

**Figure 2:**
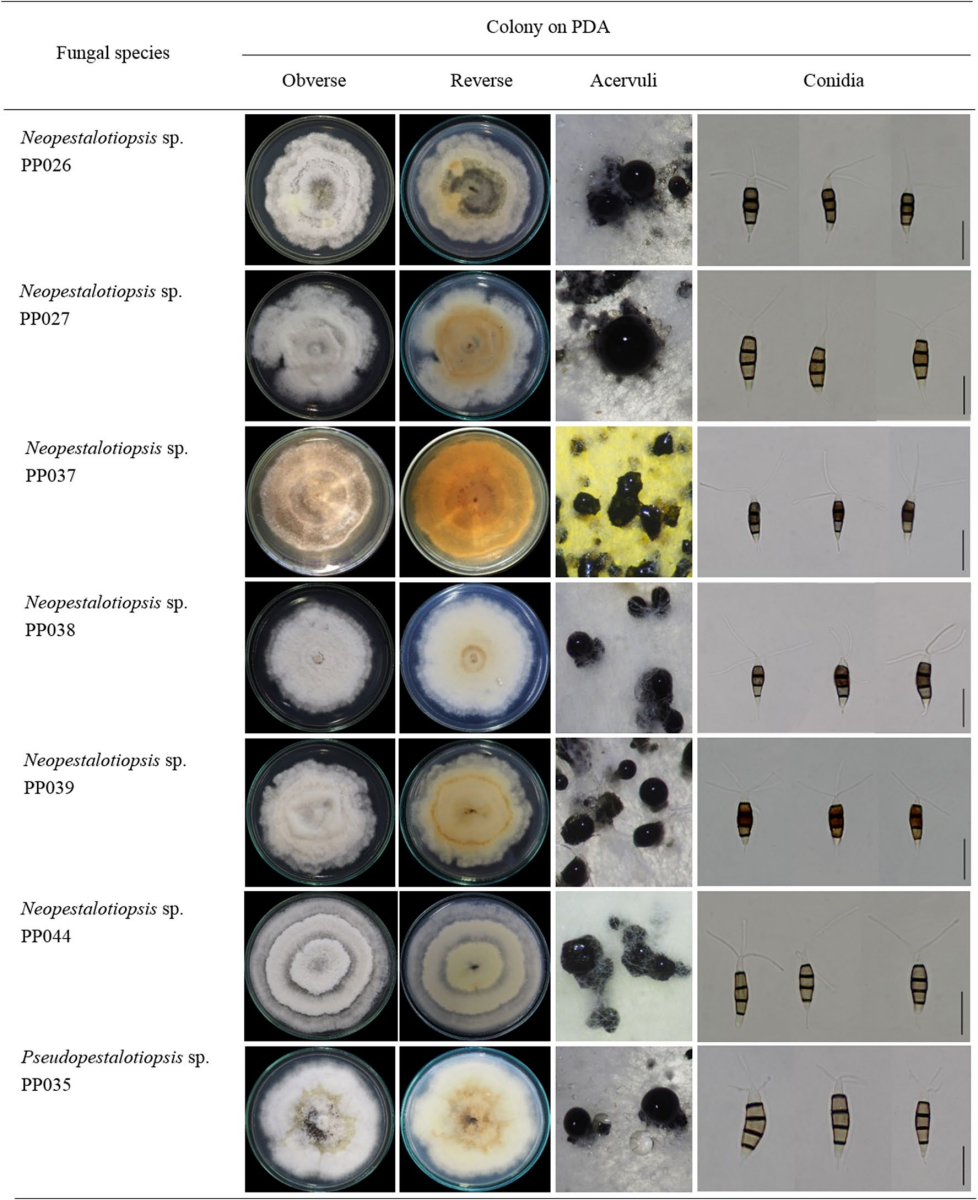
Morphological characteristics of *Neopestalotiopsis* and *Pseudopestalotiopsis* species cultivated on PDA for 7 days at 25 ± 2 °C. Scale bars represent 20 µm.

In total, 44 fungi were isolated from eight diseased plant samples, as shown in Fig. 1 and were classified into pestalotioid taxa based on morphological characteristics. Of the isolates, 2, 5, 15, 1, 6, 9, 1, and 5 were derived from jackfruit, rose apple, mangosteen, plum, snake fruit, rambutan, strawberry, and avocado, respectively (Table 1). All isolates were cultivated on PDA and maintained as axenic cultures using the specimen codes PP001–PP044. The most representative characteristic features of colony morphology and the acervuli and conidia were similar in all species. The characteristic lightly concolorous, darkly concolorous, or versicolorous appearance of conidia is considered a key character for identifying fungal species. Of the 44 isolates, 7 distinct morphological characteristics were observed (PP026, PP027, PP035, PP037, PP038, PP039, and PP044), as shown in Fig. 2.

**Figure 3:**
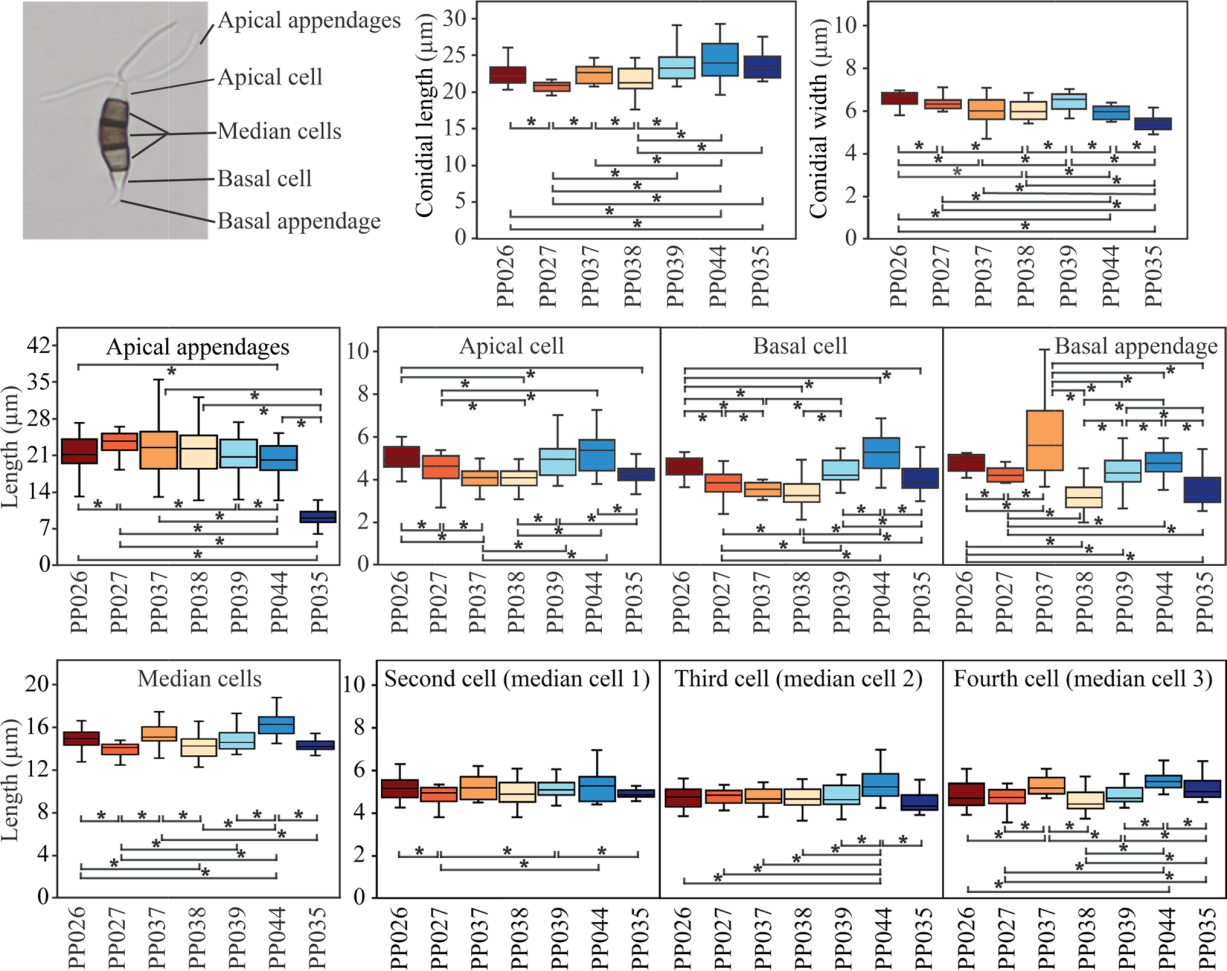
Conidial sizes of *Neopestalotiopsis* (PP026, PP027, PP037, PP038, PP039, and PP044) and *Pseudopestalotiopsis* (PP035). The upper-left corner represents conidial cells that are measured. Box plots are from the measurements of 20 conidia per isolate. Brackets with asterisks denote pairs differing at P < 0.05.

These seven isolates showed different colony patterns on PDA plates, and many had an undulate edge. PP037 produced yellowish aerial mycelia while others displayed whitish mycelia. The colony diameters of PP026, PP027, P035, PP037, PP038, PP039, and PP044 after incubating at 28 °C for 7 days were 76, 81, 83, 83, 70, 65, and 90 mm, respectively. All isolates produced black fruiting bodies or acervuli. Conidia were fusiform with 2–3 apical appendages and 1 basal appendage. Conidial lengths of all isolates varied between about 18 to almost 30 µm (Fig. 3). Conidial widths were about 4.5–7.3 µm. Cells of the conidia and appendages of each isolate were designated as apical appendages, apical cell, three median cells, basal cell, and basal appendage and measured for length accordingly. The median cell of all isolates was approximately similar with lengths of 11.7–18.9 µm. Apical and basal cells were around 2.5–7 µm. The apical appendages of PP035 were 7.7–19.8 µm while the other six isolates were extended to 35.4 µm. Basal appendages ranged from 2 to 11.5 µm.

**Figure 4:**
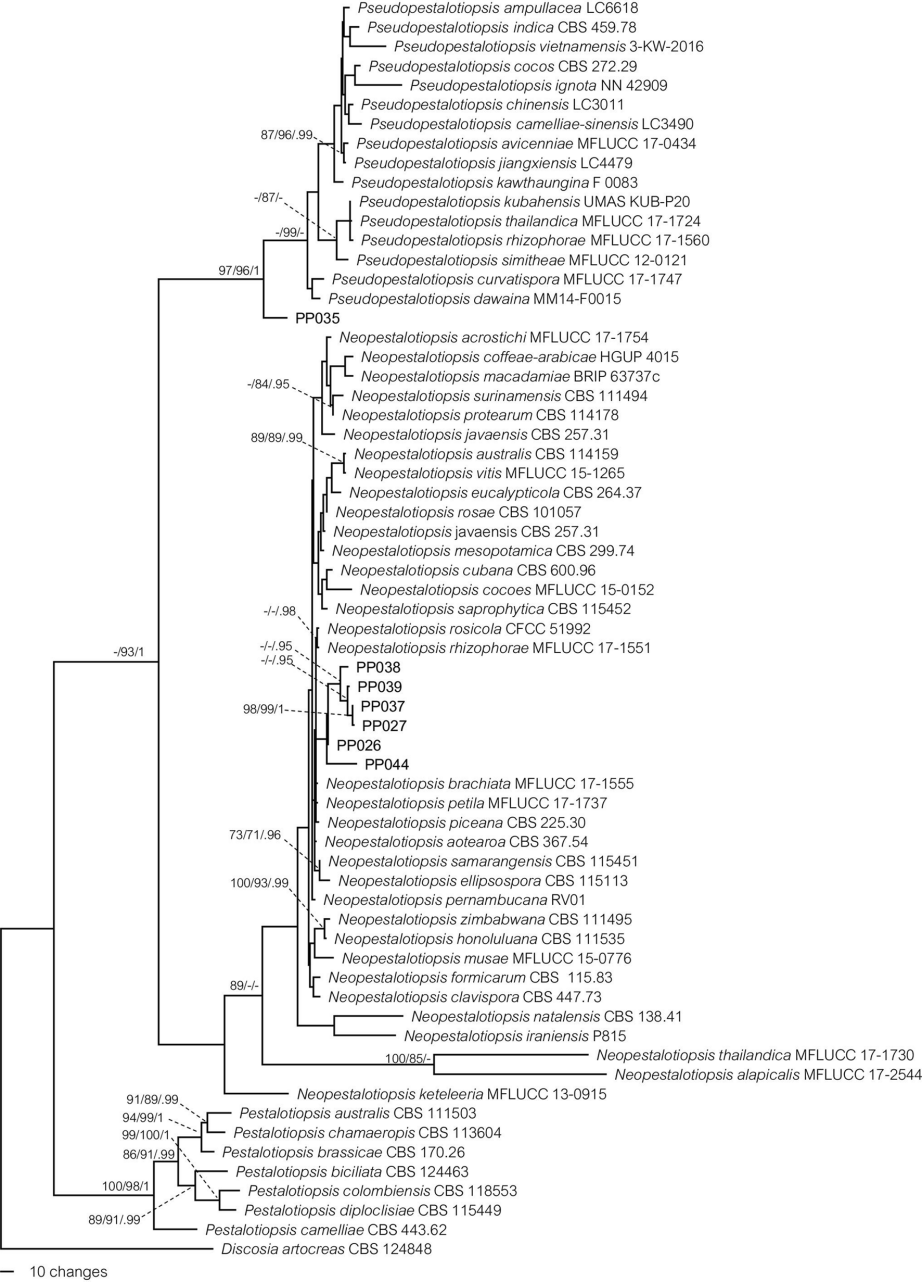
MP tree based on analysis of combined ITS, β-tubulin, and *tef1* dataset. *Discosia artocreas* (CBS124848) was used as the outgroup taxon, and new isolates are in bold. Bootstrap support values for Maximum Parsimony (MP-BS) and Maximum Likelihood (ML-BS) smaller than 70% (left and middle) are not shown and Bayesian posterior probabilities (BPP) equal to, or higher than 0.95 PP (right) are indicated at the nodes (MP-BS/ML-BS/BPP).

Seven distinct characteristics were considered for molecular analysis using multilocus sequences and six isolates were identified as *Neopestalotiopsis* spp. and one was *Pseudopestalotiopsis* sp. For phylogenetic analyses, the alignment for *Pseudopestalotiopsis* and *Neopestalotiopsis* consisted of 65 taxa, with *Discosia artocreas* (CBS124848) as the outgroup taxon. The alignment comprised 1622 characters of which 345 were variable, 752 were constant, and 525 were parsimony-informative characters, including gaps (ITS: 1–430, *tef1*: 431–892, and β-tubulin: 893–1622). The parsimony analysis resulted in three equally parsimonious trees and the first tree (TL = 2114, CI = 0.597, HI = 0.403, RI = 0.823, and RC = 0.492) was selected, as shown in Fig. 4. Phylogenetic analyses with MP, ML, and BI using the dataset of individual loci were also performed, but no topological phylogenetic conflict was observed (data not shown). Multilocus phylogenetic analyses with our fungal isolates and those for representative *Neopestalotiopsis*, *Pseudopestalotiopsis* species retrieved from the NCBI GenBank database were separated into two clades, with an ML bootstrap value of 93%. Six of the isolates (PP026, PP027, PP037, PP038, PP039, and PP044) were clustered with *Neopestalotiopsis*, while PP035 was sister to *Pseudopestalotiopsis* isolates (MP-BS = 97%, ML-BS = 96%, and BPP = 1). In this study, the phylogenetic analyses revealed that our representative isolates were supported by moderate to high bootstrap values and were consistent with the morphology-based classification. Based on the morphological and molecular evidence, our results indicated that these seven isolates were candidates for two species, namely *Neopestalotiopsis* spp. (PP026, PP027, PP037, PP038, PP039, and PP044) and *Pseudopestalotiopsis* sp. and (PP035).

### Pathogenicity and cross-inoculation

**Figure 5:**
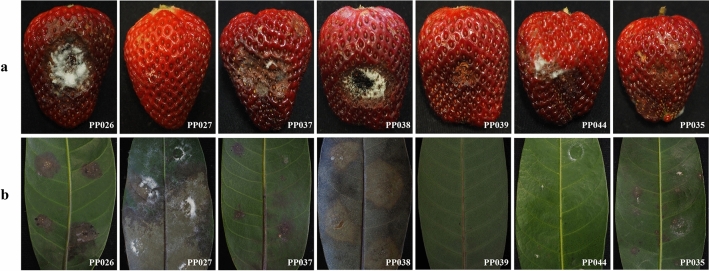
Pathogenicity of *Neopestalotiopsis* (PP026, PP027, PP037, PP038, PP039, and PP044) and *Pseudopestalotiopsis* (PP035) isolates on (**a**) strawberry fruits and (**b**) plum leaves.

Since *Neopestalotiopsis* and *Pseudopestalotiopsis* are known to have a wide host range, we investigated whether the isolates in this study could cross infect a range of fruit species. Six *Neopestalotiopsis* (PP026, PP027, PP037, PP038, PP039, and PP044) and one *Pseudopestalotiopsis* (PP035) were tested to evaluate the effect of cross-inoculation on eight plant species including their original hosts. Light brown lesions surrounded by a dark brown border, which resembled symptoms that occurred in the field, were observed on the leaves and fruits at 3–7 days after inoculation, while these symptoms did not occur in the control inoculated with agar media. The development of a necrotic zone or a lesion of at least 2.0 mm beyond the inoculation site was considered to indicate a positive status of the plant hosts. The results revealed that *Neopestalotiopsis* PP026 had a wide host range since it caused symptoms on seven fruit species except for avocado, while PP039 and PP044 infected only two fruit species and neither of them was the original host (Table 2). PP027 did not produce symptoms on the original host. Strawberry was highly susceptible to six pestalotioid isolates, except for PP027 (Fig. 5a). Plum ranked second for susceptibility, since it showed symptoms of inoculation with five pestalotioid isolates (Fig. 5b). Conversely, snake fruit displayed inoculation symptoms only with *Pseudopestalotiopsis* and not with *Neopestalotiopsis*.

**Table 2:** Pathogenicity and cross-inoculation of *Neopestalotiopsis* and *Pseudopestalotiopsis* isolates on different tropical fruit hosts.

Fungal isolate	Original host	Cross-inoculation host							
		Mangosteen	Rambutan	Jackfruit	Strawberry	Rose apple	Plum	Snake fruit	Avocado
PP026 *Neopestalotiopsis* sp.	Mangosteen	**+**	**+**	**+**	**+**	**+**	**+**	**−**	**+**
PP027 *Neopestalotiopsis* sp.	Rambutan	**+**	**+**	**+**	**−**	**−**	**+**	**−**	**+**
PP037 *Neopestalotiopsis* sp.	Jackfruit	**+**	**−**	**−**	**+**	**−**	**+**	**−**	**−**
PP038 *Neopestalotiopsis* sp.	Strawberry	**−**	**−**	**−**	**+**	**+**	**+**	**−**	**−**
PP039 *Neopestalotiopsis* sp.	Rose apple	**−**	**−**	**+**	**+**	**−**	**−**	**−**	**−**
PP044 *Neopestalotiopsis* sp.	Plum	**−**	**−**	**−**	**+**	**−**	**−**	**−**	**+**
PP035 *Pseudopestalotiopsis* sp.	Snake fruit	**+**	**−**	**−**	**+**	**−**	**+**	**+**	**−**

**+ **Observed symptoms.

**−** No symptoms.

### Effect of crude plant extracts and fungicides on mycelial inhibition

**Figure 6:**
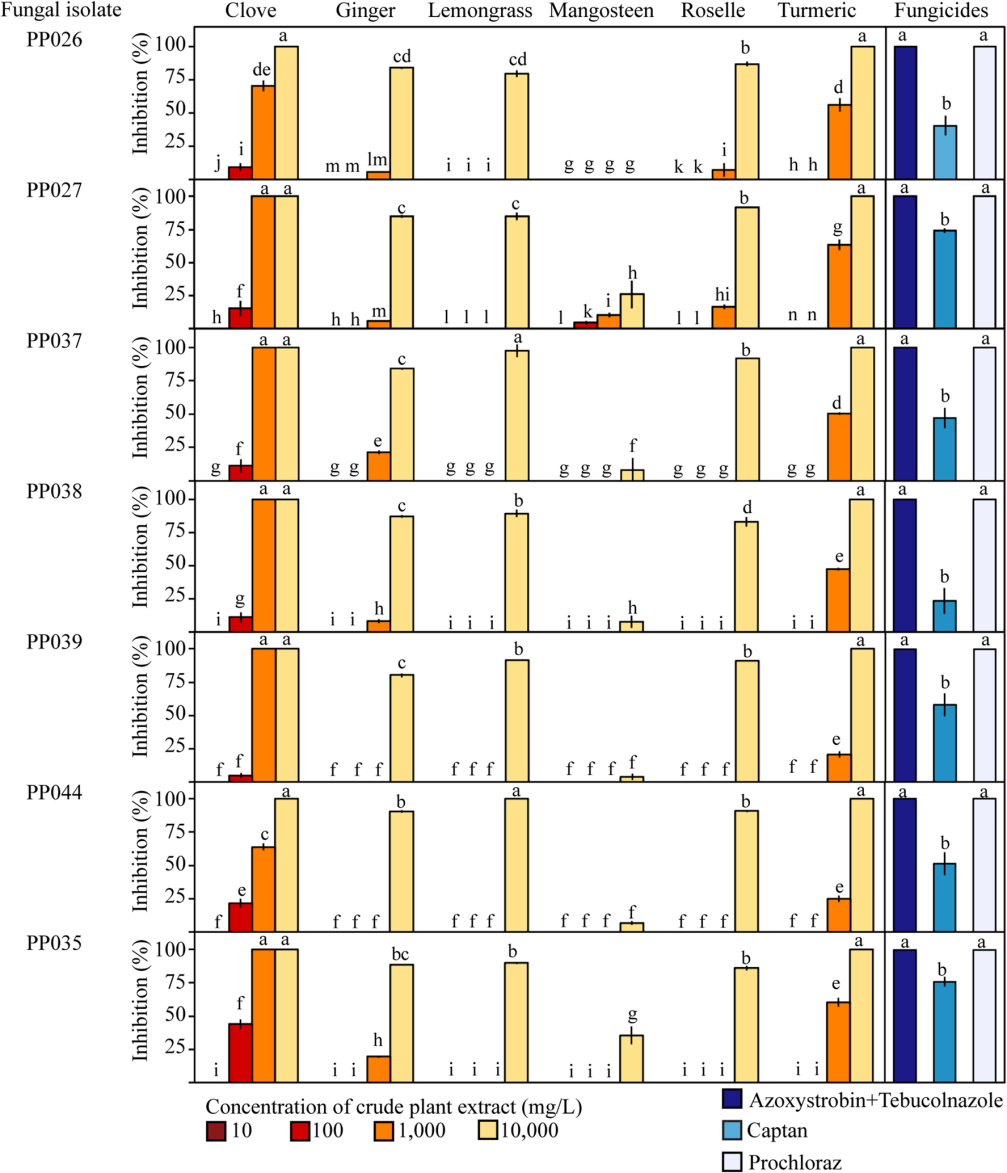
Effect of six crude plant extracts (clove, ginger, lemongrass, mangosteen, roselle, and turmeric) at concentrations of 10, 100, 1,000, and 10,000 mg/L on mycelial growth inhibition of *Neopestalotiopsis* (PP026, PP027, PP037, PP038, PP039, and PP044) and *Pseudopestalotiopsis* (PP035) compared to inhibition of azoxystrobin + tebucolnazole, captan, or prochloraz fungicides. Same lowercase letters represent no significant difference (P < 0.05).

Six crude plant extracts from clove, ginger, lemongrass, mangosteen, roselle, and turmeric were studied for their ability to inhibit the growth of *Neopestalotiopsis* and *Pseudopestalotiopsis* and compared with fungicide treatments for controls. The results showed that at the highest concentrations (10,000 mg/l), the clove and turmeric crude extracts completely inhibited the growth of all isolates after incubation for 14 days*,* followed by the effects of ginger, lemongrass, and roselle extracts, whereas the crude extract of mangosteen exhibited comparatively very low activity against all isolates (Fig. 6). Although clove extracts at 1000 mg/l displayed some inhibition, the other crude plant extracts with lower concentrations did not exhibit antifungal activity against the *Pseudopestalotiopsis* or *Neopestalotiopsis* species. To compare the effectiveness of crude plant extracts with chemical treatments, three fungicides (azoxystrobin + tebucolnazole, captan, or prochloraz) were tested at the recommended dose. Azoxystrobin + tebucolnazole and prochloraz both showed 100% inhibition of the mycelial growth of all *Neopestalotiopsis* and *Pseudopestalotiopsis* isolates, while captan exhibited less effectiveness.

## Discussion

The genus *Pestalotiopsis* was previously separated from the genus *Pestalotia* and classified in the Ascomycota, Coleomycetes, as proposed by Barr^48^. In the present study, a new re-classification of *Pestalotiopsis* was performed including two other genera. The genus *Neopestalotiopsis* spp. is characterized by versicolorous median cells, *Pestalotiopsis* spp. is characterized by lightly pigmented concolorous median cells, and *Pseudopestalotiopsis* spp. are characterized by darkly concolorous median cells^1^.

*Neopestalotiopsis* and *Pseudopestalotiopsis* are not highly host-specific, and these taxa may have the ability to infect a range of hosts^49, 50^. In this study, all tested isolates could infect the surface tissue of many plants based on the wounding inoculation method, which avoids any interference caused by host penetration resistance. Typical symptoms were observed including the browning of foliage, leaf blight, fruit rot, and the presence of black acervuli on diseased tissue. However, some fungal species failed to infect other inoculated hosts that might have been caused by environmental conditions, especially the temperature of the room used to incubate the inoculated fruits, which might have been too high for these genera exhibiting an optimum temperature of 22–28 °C ^51^ and optimal relative humidity ranging from 65 to 100%^49^.

Examination of the efficacy of the six plant extracts against *Neopestalotiopsis* and *Pseudopestalotiopsis* species showed that crude extracts from clove and turmeric had the highest antifungal efficacy against all the tested isolates. The antimicrobial properties of clove and turmeric have been well established, especially against bacterial and fungal pathogens*.* These two species produce active agents such as curcumin, eugenol, and eugenyl acetate, which are effective against bacteria and phytopathogenic fungi^28, 29^. Roselle, lemongrass, and ginger extracts are also reported to possess antibacterial and antifungal properties against pathogens^26, 27, 30^, while mangosteen are not effective. Although xanthones in mangosteen affect *Alternaria solani*, *Aspergillus flavus*, and *Fusarium* spp.^52^, mangosteen extracts display comparatively very low activity against *Colletotrichum gloeosporioides*^53^.

Crude plant extracts are comparable to fungicides to control *Neopestalotiopsis* and *Pseudopestalotiopsis*. Clove and turmeric extracts on the inhibition of mycelial growth of pestalotioid fungi are equivalent to the efficiency of azoxystrobin + tebucolnazole or prochloraz, which had 100% inhibition. Similarly, azoxystrobin and prochloraz can inhibit *P. microspora*, which causes leaf spot disease of *Photinia fraseri* in China^54^. The present study showed that captan inhibited 20–80% of the growth of pestalotioid fungi. Similarly, Esiegbuya et al.^55^ reported the effect of captan at 250 and 500 ppm on the in vitro mycelial growth of *P. clavispora;* however, the fungicide did not completely inhibit the pathogen*.*

Therefore, it was assumed that the antifungal properties of these plant extracts may be principally due to the presence of unknown compounds that could not be analyzed and identified in the present study. Hence, further study should investigate the identification of the active compounds of plant extracts that control all pathogens, and the tested plants may be used for developing new fungicides that are safe for consumers and farmers and are effective in the field.

## Data Availability

Sequence data that support the findings of this study have been deposited in GenBank with the accession numbers: MT628375–MT628381, MW776648–MW776654, and MW776633–MW776638.
